# A resting state network in the motor control circuit of the basal ganglia

**DOI:** 10.1186/1471-2202-10-137

**Published:** 2009-11-23

**Authors:** Simon Robinson, Gianpaolo Basso, Nicola Soldati, Uta Sailer, Jorge Jovicich, Lorenzo Bruzzone, Ilse Kryspin-Exner, Herbert Bauer, Ewald Moser

**Affiliations:** 1 Functional Neuroimaging Laboratory, Center for Mind/Brain Sciences, University of Trento, Trento, Italy; 2 Telecommunication Engineering, University of Trento, Trento, Italy; 3 Faculty of Psychology, University of Vienna, Liebiggasse 5, 1010 Vienna, Austria; 4 MR Center of Excellence, Medical University of Vienna, Lazarettgasse 14, 1090 Vienna, Austria; 5 Center for Biomedical Engineering and Physics, Medical University of Vienna, Währinger Gürtel 18-20, 1090 Vienna, Austria

## Abstract

**Background:**

In the absence of overt stimuli, the brain shows correlated fluctuations in functionally related brain regions. Approximately ten largely independent resting state networks (RSNs) showing this behaviour have been documented to date. Recent studies have reported the existence of an RSN in the basal ganglia - albeit inconsistently and without the means to interpret its function. Using two large study groups with different resting state conditions and MR protocols, the reproducibility of the network across subjects, behavioural conditions and acquisition parameters is assessed. Independent Component Analysis (ICA), combined with novel analyses of temporal features, is applied to establish the basis of signal fluctuations in the network and its relation to other RSNs. Reference to prior probabilistic diffusion tractography work is used to identify the basal ganglia circuit to which these fluctuations correspond.

**Results:**

An RSN is identified in the basal ganglia and thalamus, comprising the pallidum, putamen, subthalamic nucleus and substantia nigra, with a projection also to the supplementary motor area. Participating nuclei and thalamo-cortical connection probabilities allow this network to be identified as the motor control circuit of the basal ganglia. The network was reproducibly identified across subjects, behavioural conditions (fixation, eyes closed), field strength and echo-planar imaging parameters. It shows a frequency peak at 0.025 ± 0.007 Hz and is most similar in spectral composition to the Default Mode (DM), a network of regions that is more active at rest than during task processing. Frequency features allow the network to be classified as an RSN rather than a physiological artefact. Fluctuations in this RSN are correlated with those in the task-positive fronto-parietal network and anticorrelated with those in the DM, whose hemodynamic response it anticipates.

**Conclusion:**

Although the basal ganglia RSN has not been reported in most ICA-based studies using a similar methodology, we demonstrate that it is reproducible across subjects, common resting state conditions and imaging parameters, and show that it corresponds with the motor control circuit. This characterisation of the basal ganglia network opens a potential means to investigate the motor-related neuropathologies in which the basal ganglia are involved.

## Background

A number of studies dating back to 1995 have shown that when subjects are not engaged in processing externally directed tasks or time-varying stimuli - when they are, from a behavioural perspective, at rest - MR images of the brain show correlated, low frequency fluctuations in functionally related areas. This has been interpreted as indicating functional connectivity between regions [1-5]. A number of distinct, largely independent assemblies, or Resting State Networks (RSNs) have been discovered since using semi-exploratory and exploratory analysis methods.

It has recently been shown that RSN fluctuations explain not only inter-trial variation in the BOLD response [6] but also behaviour [7] and that some RSNs are disturbed in pathologies such as Alzheimer's disease (e.g. [8]). This has fuelled efforts to better characterise these networks through behavioural manipulation [9,10] and by their interdependence on other networks [11,12], not only to improve experiment design but also to better understand healthy brain function and a range of neurological and psychiatric conditions.

The first RSNs were discovered using functional connectivity analysis, in which correlation is performed between the time course in a seed voxel or region and that in other voxels, in order to reveal areas whose activity is coupled. The discovery that functional connectivity could be observed between ipsilateral and contralateral sensorimotor regions [1] was rapidly followed by similar observations for visual and auditory areas [13], the amygdala [4] and the thalamus and hippocampus [14]. It was later discovered that the group of regions which have come to be known as the Default Mode network, which had been observed to be more active during rest periods than during task processing [15-17], also show fluctuations characteristic of RSNs during rest periods [5]. The development of group Independent Component Analysis methods allowed a fully exploratory approach to identifying RSNs [18-20], and led to the elucidation of other networks in posterior parietal areas, lateralised left and right frontoparietal regions, the anterior temporal lobe, cerebellum and limbic lobe [9,21-23]. To date, approximately 10 RSNs have been reproducibly identified [9,23].

There is no a priori model in functional connectivity analysis, but a seed voxel (or ROI) time-course is selected by the experimenter. This process leaves the approach prone to omission unless correlations are computed between a large number of regions (see, e.g., Achard et al. [24]), and also to weakening by inter-subject variation if seed regions are defined on the basis of template anatomy rather than individual function. Activation results from functional tasks may be used to define seed regions, but this becomes impractical if many networks are to be analysed in the same data. The sensitivity of the analysis is reduced if sub-regions of the same network are separated according to a hypothesis about possible division of function. Alternatively, erroneous conclusions may be drawn about regions functionally connected if seed regions are used that subsume areas which contribute to different networks. In addition, the signal in a seed region comprises many sources, of both neuronal and non-neuronal origin (such as scanner drift). While the inclusion of motion parameters in the general linear model as well as regressors for global, ventricular and white matter signals allows these effects to be reduced [11,25], it has proved difficult to separate a number of physiological artefacts from RSN-related fluctuations, such as those arising from changes in respiratory rate from the Default Mode [26]. Likewise, the subtraction of global signal to try to mitigate this problem may introduce artificial anticorrelation relationships between component time courses [25,27,28], making study of the interrelation between networks problematic. As a genuinely exploratory method, ICA yields potentially interesting components in the context of the other signal fluctuations in the data and is generally capable of separating overlapping signal sources of physiological and neuronal origin [29]. As such is it well suited to an exploratory analysis aimed of RSNs and their interrelations.

Most RSN findings to date relate to the cerebral cortex. There are reports, mostly restricted to the functional connectivity literature, of correlated fluctuations between isolated subcortical structures, including the amygdala [4] and the thalamus and hippocampus [14]. The involvement of the hippocampus in a (usually subdivided) sensory-auditory RSN [22] and in the Default Mode has been noted [22] as has the inclusion of number of thalamic nuclei in the medial visual, auditory and medial frontal RSNs [21]. Jafri et al. also identify elements of the basal ganglia, albeit in a predominantly cortical frontal parietal subcortical network [12]. We recently reported the existence of an RSN in the basal ganglia and thalamus with weak projections to supplementary motor areas [30]. This network overlaps substantially with that identified in a contemporaneous functional connectivity study by Di Martino et al. (corresponding to seeds in the dorsal and caudal putamen) [31], and a subsequent incidental noting of the same network by Damoiseaux et al. [32]. The network was not present in any other ICA-based study of which we are aware (e.g. [9,21-23]).

These scant and inconsistent reports leave the consistency and role of this network open to question. A recent dispute indicates that apparently subtle variations in behavioural condition can give rise to the appearance of spontaneous activation [33]. Both the Di Martino et al. study [31] and our initial report [30] were based on subjects visually fixating, unlike the majority of RSN studies, in which subjects had their eyes closed, and in which the basal ganglia RSN was not observed. In that light, it seems pertinent to investigate the conditions under which this network is manifest. The origin of these signal fluctuations likewise needs to be established; whether they are neuronal resting-state fluctuations or a physiological artefact. If the origins of these network are neuronal, to which of the many parallel basal ganglia circuits and functions do they correspond, and what relationship do they have to other resting state networks?

The basal ganglia consist of four nuclei; the striatum (which is subdivided into the caudate nucleus and putamen), the globus pallidus or pallidum, the substantia nigra and the subthalamic nucleus [34]. Originally viewed as motor structures, tracing studies in the monkey suggested that the striatum could be divided into two networks, sensorimotor and associative [35]. A more recent model has suggested that the diverse functions in which the basal ganglia are involved are expressed through five parallel segregated circuits; motor, oculomotor, dorsolateral prefrontal, lateral orbitofrontal and anterior cingulate [36]. Each of these receives input from a number of functionally related neocortical regions (e.g. in the case of the motor circuit, the supplementary motor area, arcuate premotor area, motor cortex and somatosensory cortex) and outputs to a single frontal region (e.g. in the motor circuit, the supplementary motor area). In the current view, the basal ganglia are envisaged as comprising just three distinct functional assemblies; the sensorimotor, the associative and the limbic [37]. The fact that each of the corresponding circuits involves distinct regions of striatum, pallidum, substantia nigra, thalamus and cortex offers the possibility - within the limits of activation localisation - of identifying the circuit to which the network corresponds, and with that, its function.

We examine resting-state fluctuations in the thalamus and basal ganglia using two common resting state conditions, two large, independent study groups and a fast EPI protocol optimised for structures with a short T2* [38,39], combined with high field strength. We apply group ICA tools to identify this network in studies using fixation and eyes closed conditions, and refer to known basal ganglia circuits and probabilistic tractography to identify the function subsumed by this network. Temporal features of independent components are used in a novel classifier to distinguish RSNs from physiological artefacts, and functional network connectivity is applied to elucidate the relationship between the basal ganglia resting state network and the Default Mode.

## Results

We find a resting state network involving the thalamus and a large portion of the basal ganglia in groups studied under both the eyes open and fixation conditions, and a temporally coherent network focussed on the caudate in the fixation study only.

**Figure 1:**
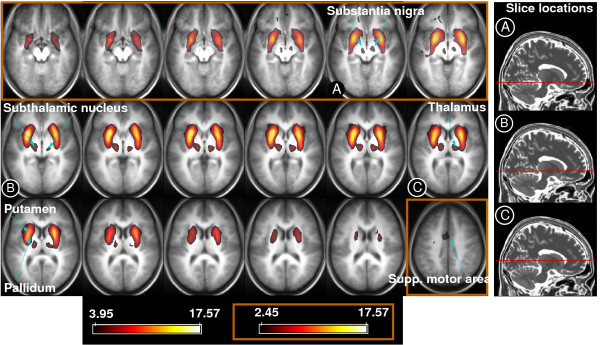
**The basal ganglia resting state network**. It comprises, bilaterally, the pallidum, putamen, substantia nigra, subthalamic nucleus and thalamus. In the overlay, which is on the mean anatomical image for the group, maps of z statistics are thresholded at P > 0.99 (see colorbar on bottom left) other than slices outlined in brown, which are thresholded at P > 0.5 (see colorbar on bottom right in brown box). All slices are shown thresholded at P > 0.5 in Additional File 1.

The distributed basal ganglia network is symmetric and incorporates the pallidum, putamen, subthalamic nucleus and substantia nigra, as well as the thalamus, with weaker extensions to the transverse temporal gyrus and the supplementary motor area (SMA). The network is illustrated as it appears in the MELODIC analysis of the fixation study group of 26 subjects in Figure 1. Previous studies employing PICA have employed a posterior probability threshold of P > 0.5 using the mixture model approach [18,21-23,32,40]. Non-outlined slices in Figure 1 derive from ICA of downsampled, smoothed data and are thresholded using a more stringent threshold of P > 0.99. To enable more exact localisation of activation foci in the region of the substantia nigra and subthalamic nucleus, results based on unsmoothed, non-resampled data have been illustrated for that region (the top row of Figure 1, slices outlined in brown), and are thresholded at the canonical level of P > 0.5. The same data and threshold were used for the slice showing activation in the SMA (Figure 1C). The component is overlaid on the mean anatomical (MPRAGE) image for the group. To aid orientation the location of selected slices (labelled A-C) is indicated on the MNI template T_2 _image (right panel). The entirety of the network is shown in Additional File 1 (downsampled data, thresholded at P > 0.5).

**Figure 2:**
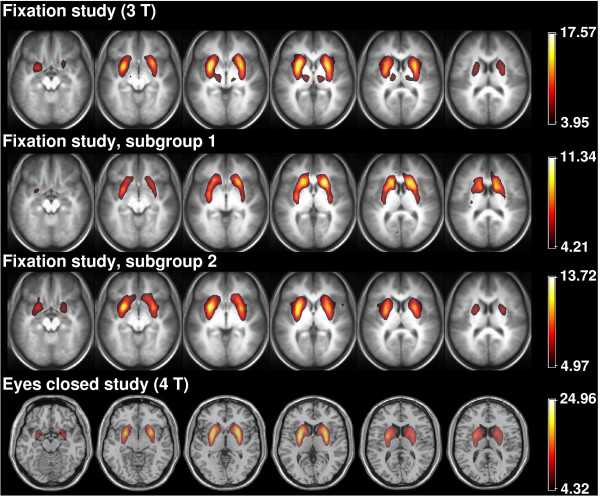
**The reproducibility of the basal ganglia resting network**. The top row shows selected slices from the main study with all subjects. The second and third rows illustrate results for subgroups 1 and 2 respectively. The bottom row shows the same network as it appears in the eyes closed study (eyes closed, 4 T, both male and female participants). All RSN z maps are thresholded at P > 0.99.

The basal ganglia network is also present in the analyses of the two subgroups of 13 subjects in the fixation study (Rows 2 and 3 respectively in Figure 2) and in the eyes closed study in which a separate group of subjects were scanned with their eyes closed (Row 4 in the same figure). In all cases, it appears substantially unvaried from its manifestation in the whole group analysis (Row 1). The eyes closed study included both male and female subjects and was conducted at 4 T with a very different EPI protocol, showing the network to be reproducible across a wide range of factors; resting state condition (fixation and eyes closed), gender and measurement parameters, including field strength and repetition time.

**Figure 3:**
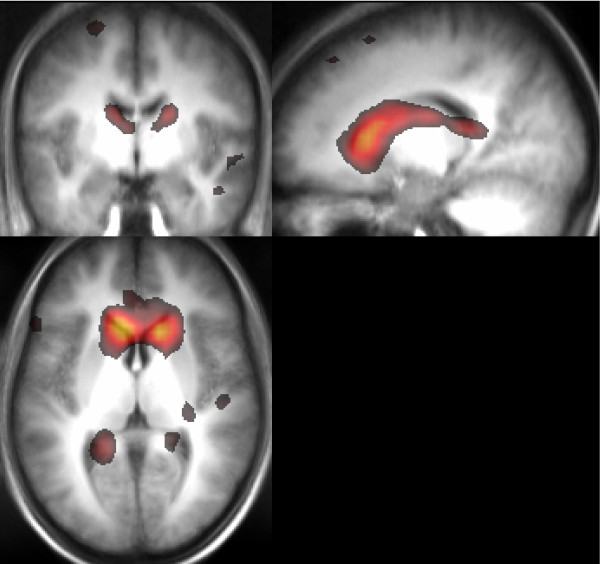
**A component showing caudate activity in the main study**. The overlay of z statistics is thresholded at P > 0.5.

In addition to the basal ganglia network, in which one element of the striatum - the caudate nucleus - is not present, a separate component was identified in the fixation study data, which consists of the caudate and, much more weakly, dorsolateral prefrontal cortex and posterior parietal cortex (Figure 3). This is absent in the study in which subjects had their eyes closed.

A public-access atlas of thalamo-cortical connection probabilities http://www.fmrib.ox.ac.uk/connect/ based on results of a prior study by Behrens et al. [41] shows that, for the basal ganglia the thalamic activation foci identified connect with high probability to motor areas. For the focus with MNI co-ordinates (13, -20, 0) these probabilities take the values of 0.68 to the primary motor, 0.44 to the sensory and 0.28 to the pre-motor cortex. For the focus at (-13, -20, 0) the respective probabilities are 0.44, 0.38 and 0.43. The probabilities of the primary thalamic connections being to none-motor regions (occipital, prefrontal, posterior parietal and temporal cortices) are below 0.2 for both foci.

All the other resting state networks reported in the ICA-based resting state literature to date [9,21-23,32] were also identified in the MELODIC analysis of the fixation study (Table 1). Despite its reproducibility, the basal ganglia network emerges as one of the weakest in these data in terms of the percentage of variance in the data explained by each component (0.13%). The only subcortical elements present in other networks were the hippocampus in the Default Mode (consistent with e.g. [15]) and the weak presence of the substantia nigra in the posterior parietal network.

**Table 1:** The RSNs identified in the MELODIC analysis of the main study group, the names adopted in this work, the relative strength of the components and correspondence to RSNs identified in other studies (the bracketed suffixes L and R indicate that the component identified here corresponds to either the left or right hand side of the network referred to).

Component Number	RSN Name	Percentage of explained variance	**Labeling in [(Calhoun et al., 2008), (Damoiseaux et al., 2006), (Beckmann et al., 2005), (De Luca et al., 2006)] respectively**.
10	Medial visual	3.81	[D, E,(a),]
11	Motor	3.59	[B,F,(d),RSN3]
12	Cerebellum	3.31	[H,,,]
15	Lateral visual	2.67	[F,A,(b),RSN1]
20	Posterior parietal	2.18	[C,H,,]
23	Left lateral fronto-parietal	1.93	[E,C,(h),RSN4(L)]
28	Temporal lobe	1.15	[I,I,(c),RSN3]
31	Medial frontal	0.85	[J,,(f),RSN2]
36	Default Mode	0.53	[A,B,(e),RSN2]
40	Limbic lobe	0.28	[L,,,]
41	Basal ganglia	0.13	
43	Right lateral fronto-parietal	0.08	[K,D,(g),RSN4(R)]
52	Anterior temporal lobe	†	[G,,,]

† - no valid value was obtained for this component.

The same resting state networks, including the basal ganglia resting state network, were identified in the GIFT results, which were used to extract single-subject temporal responses for the assessments of temporal and frequency features that follow. The spatial map of the basal ganglia component identified in the GIFT analysis is shown in Additional File 2.

**Figure 4:**
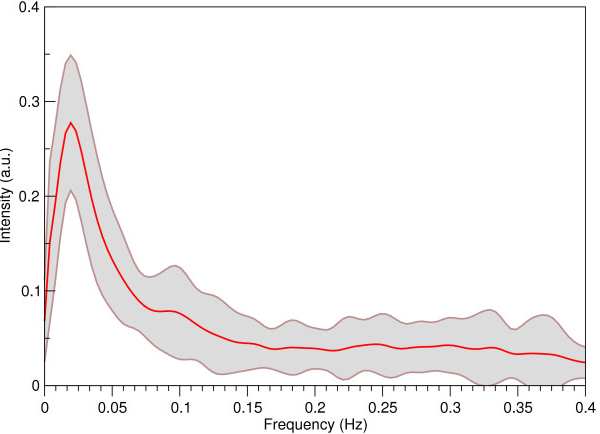
**Mean frequency spectrum of the network (over subjects)**. The boundaries of the shaded area denote one standard deviation on the mean.

The mean frequency spectrum of the basal ganglia network is illustrated in Figure 4, in which the grey band indicates one standard deviation from the mean across subjects. On average, the position of the maximum in the spectral distribution was at 0.025 ± 0.007 Hz.

To investigate the possibility of causal relationships existing between the basal ganglia and other RSNs (similar to those which have been demonstrated between the Default Mode and the lateral parietal networks [5,11,42,43]), the mean correlation was calculated between component time courses for the basal ganglia RSN and other RSNs over all subjects. The most significant results are listed in Table 2. The basal ganglia RSN was anti-correlated with the Default Mode (-0.27 ± 0.24, p = 8.4 × 10^-6^) and positively correlated, to a similar degree, with both the right and left lateral fronto-parietal networks (0.20 ± 0.26, p = 5.8 × 10^-4 ^and 0.24 ± 0.18, p = 9.4 × 10^-7^, respectively). These values may be compared with those obtained in this analysis between the Default Mode and the lateral fronto-parietal networks; -0.19 ± 0.34, p = 0.011 (right) and -0.19 ± 0.21, p = 1.6 × 10^-4 ^(left). That is, the correlations between the basal ganglia RSN and the Default Mode, and the basal ganglia RSN and lateral parietal networks are consistent with those between the Default Mode and the lateral parietal networks but are stronger and more significant. Functional network connectivity analysis [12] applied to the basal ganglia RSN and the Default mode - performed for 20 randomly-composed groups of 13 subjects from the population of 26 subjects - showed significant correlation between the two networks for all 20 groups. In 18 of these the hemodynamic response in the basal ganglia was in anticipation of that in the Default Mode (by an average of 0.56 ± 0.51 s), in 2 it lagged behind it.

**Table 2:** Mean correlation between the basal ganglia component time course and the time courses of other networks, across subjects, listing the most significant mean correlations.

Network Names	Correlation	Standard deviation	P
Basal ganglia, Default Mode	-0.27	0.24	8.4 × 10^-6^
Basal ganglia, right lateral fronto-parietal	0.20	0.26	5.8 × 10^-4^
Basal ganglia, left lateral fronto-parietal	0.24	0.18	9.4 × 10^-7^

*Default Mode, right lateral fronto-parietal	-0.19	0.29	0.011
*Default Mode, left lateral fronto-parietal	-0.19	0.34	1.6 × 10^-4^

*These networks possess an anti-correlation well-documented in the literature, and are presented for comparison.

**Figure 5:**
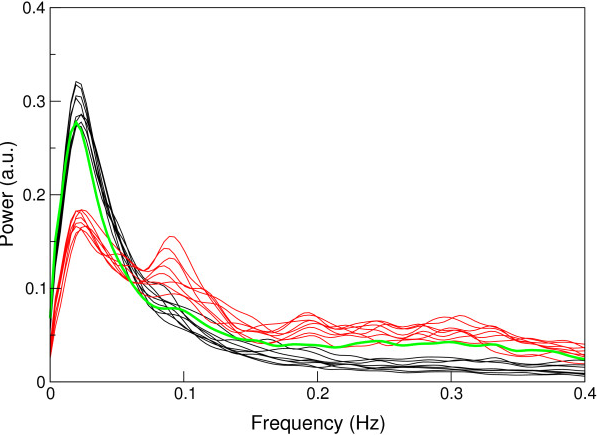
**Comparison of the mean spectral distribution of the basal ganglia RSN component over subjects (green) with the spectral distributions of documented RSNs (black) and physiological components (red)**.

**Figure 6:**
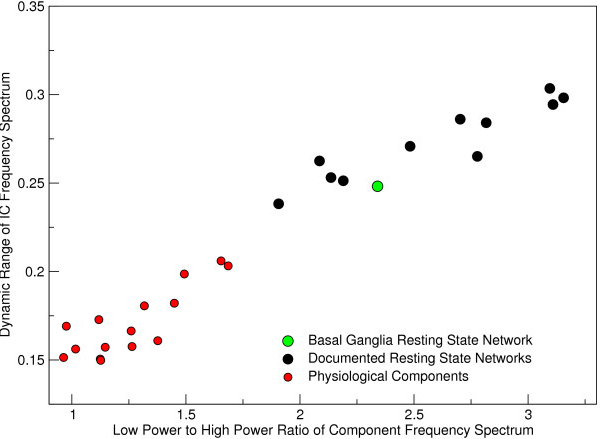
**Plot of the space of the spectral features described in the text, indicating separation of the RSNs documented to date (black) from components reflecting physiological signal fluctuations (red)**. The frequency properties of the basal ganglia (green) show it to group with other RSNs.

A number of components were identified by experienced raters (SR and GB) as being of physiological origin based on their spatial distribution. These included components of vascular origin located in the Circle of Willis, distributed grey matter components arising from respiration rate variation of the type reported by Birn et al. [29], and CSF flow between the ventricles. The mean frequency spectrum of the basal ganglia RSN is also plotted in Figure 5 along with the frequency spectra of components of established RSN origin and physiological components. Spectral power was focussed at lower frequencies for RSNs than physiological components. The two frequency features described in the Methods section, "Dynamic Range" and "Low to High Power Ratio", are plotted for RSNs and physiological component spectra in Figure 6. The known RSNs are well separated from physiological components. The basal ganglia component clusters with known RSNs.

The "Dynamic Range" feature afforded 93% accuracy in distinguishing resting state networks and physiological components, with 0 false negatives (no RSNs incorrectly identified as a physiological signal source) and 1 false positive (1 physiological component labelled incorrectly as an RSN). The basal ganglia component was classified as an RSN.

## Discussion

We detail the structures contributing to a recently reported resting state network in the thalamus and basal ganglia. By using a high field, high BOLD sensitivity experiment design and high resolution analysis, we show that it encompasses not only the thalamus, pallidum, putamen and transverse temporal gyrus - as has been previously noted - but also the substantia nigra and subthalamic nucleus, allowing the basal ganglia circuit to which it corresponds to be identified. Despite its non-observation in most resting-state studies to date [9,21-23] it was found to be reproducible across subjects and MR measurement parameters and is evident in both the eyes closed and fixation resting state conditions. The network is positively correlated with the left and right lateral fronto-parietal (also called attention or task-positive) networks, and anticorrelated with the Default Mode, whose hemodynamic response it anticipates. The peak frequency and spectral characteristics are similar to those of other RSNs, distinct from physiological fluctuations, and allow it to be classified as an RSN using the unsupervised classifier described. We proceed to identify which of the parallel segregated basal ganglia circuits this network corresponds to based on participating regions.

Because of their historical significance as motor structures, the best studied basal ganglia network is the motor circuit. Cortical input from precentral motor areas, postcentral somatosensory areas, the arcuate premotor area and the supplementary motor areas projects dominantly to the putamen. The putamen sends projections to the interior segment of the pallidum and on to particular thalamic nuclei (the direct pathway) as well as the internal segment of the globus pallidus via the caudolateral substantia nigra to the thalamus (the indirect pathway). Outputs from the thalamus project to the supplementary motor area, the motor cortex and arcuate premotor area, probably in distinct subcircuits [34]. The observed resting state network corresponds well with the motor circuit; the caudate is notably absent, and the putamen constitutes the focus of activation. The activated regions identified as substantia nigra are consistent with coordinates derived from stereotactic electrophysiological studies [44]. The presence of the substantia nigra suggests that the resting state network corresponds either solely to the indirect pathway of the motor circuit, which acts to inhibit movement, or both the indirect and direct pathways. Weak activation in the supplementary motor area is likewise consistent with the motor circuit. Myeloarchitectonics suggest that the thalamic activation corresponds to the ventro-lateral thalamic nucleus, which a prior DTI study has shown to connect dominantly to motor regions [41]. In fact, endogenous BOLD fluctuations in this part of the thalamus have been found to be strongly correlated with motor and premotor areas by Zhang et al. ([45] - supplementary material) as well as the whole putamen.

As well as there being good agreement between the areas observed in this RSN and the motor circuit, there is disparity between the principle sites of input to the striatum and the other circuits. The caudate provides input to the oculomotor, dorsolateral prefrontal and lateral orbitofrontal circuits and the ventral striatum provides input to the anterior cingulate circuit. The cortical regions to which the circuits send output are the frontal eye fields for the oculomotor circuit and the dorsolateral prefrontal cortex, the lateral orbitofrontal cortex and the anterior cingulate area for those circuits respectively.

No components were identified corresponding to the associative and limbic thalamo-cortical loops [36]. The question arises of whether it is to be expected that an RSN exists for each of the tripartite or Alexander subdivisions of the basal ganglia, and whether basal ganglia RSNs (if present) would be expected to correspond exactly with circuits that have been established from anatomical and afferent projections from the cortex. Looking outside the basal ganglia, we know that whilst RSNs reflect functional task networks, it is not the case that there is an RSN for every functional network defined by task or anatomy. Where there are spontaneous fluctuations in networks at rest, other studies have shown that there is not complete correspondence with the network as defined by anatomy or tasks. The major anatomical afferents from the cortex suggest a functional partitioning of the putamen, with a minor portion classified as associative and limbic, and the majority as being sensorimotor. Zhang et al. found functional connectivity between the motor and premotor cortex and almost the entire putamen [45], however, as observed here. The temporal cortex also displayed a much weaker correlation with the caudal-ventral putaminal region, a finding not consistent with the classical striate nucleus tripartite subdivision. In addition, when looking at RSNs involving associative cortical area, such as the Default Mode, ventral attention, dorsal attention and executive control networks, only a portion of the caudate nucleus participates, instead of the entire associative subdivision of the striate nucleus.

Our hybrid simulations suggest that ICA is capable of separating circuits which overlap to some extent, but which also have non-overlapping elements and different temporal behaviour (Additional File 3). Further evidence of this is provided by the fact that some brain regions (such as the thalamus) are present in multiple networks. The most likely explanations for not finding RSNs corresponding to the associative and limbic thalamo-cortical circuits is, therefore, that they either do not show spontaneous low-frequency BOLD fluctuations or that these fluctuations are below the sensitivity of this study.

Di Martino et al. have reported a resting state functional connectivity analysis focussing on the striatum [31] independent of our preliminary reporting of these results [30]. A gender-mixed group of subjects were studied while fixating on the word "Relax" in a study with a sensitivity likely to be equivalent to that here; applying a similar, relatively short TE EPI protocol and comparable number of total image volumes (6895 c.f. 7800 here). The connectivity results obtained for seeds in the dorsal and caudal putamen in the Di Martino et al. study is similar to the RSN observed by us [30], although the involvement of the substantia nigra (which contributes substantially to the motor circuit attribution) was not reported. Also, the dorsal putamen seeds indicated correlated activity in the anterior cortex cingulate, which relate to executive function, indicating some mixing of fluctuations relating to motor and executive control circuits. The basal ganglia RSN reported here was recently noted as an incidental finding by Damoiseaux et al. [32], adding independent verification of these results.

The conditions under which this resting state data were acquired were similar to those used in a number of previous studies which also applied group ICA approaches [9,21-23]. The question then arises as to why this network was not reported in those studies, but has been observed only here and in the most recent publication by Damoiseaux et al. [32]. The basal ganglia resting state network was one of the weakest identified in this study, measured in terms of the percentage of total variance in the data it explains. The sensitivity of this study is likely to be higher than that in the studies cited due to the high field strength (3 T), relatively large number of subjects (26) and short repetition time (1 s). The echo time of 28 ms is quite short and well matched to the T_2_* of basal ganglia structures at 3 T [38], yielding optimal BOLD sensitivity. T_2_* is shorter in the basal ganglia than is typical in the cortex due high iron concentration [46]. While it is likely that, other than reference [32], previous studies were not sufficiently sensitive to detect this network, it is also possible that it was simply overlooked in the wealth of components.

Previous studies using functional connectivity analyses have demonstrated an anticorrelation relationship between the Default Mode and the dorsal attention network in the resting state [5,11,42,43], which has been interpreted as indicating an interplay between modes thought to reflect stimulus-independent thought and goal-driven activity. These anticorrelation findings have been recently called into question, however, as global signal subtraction performed as a pre-processing step to reduce the influence of physiological noise and scanner drift itself introduces anticorrelations [27,28]. The approach taken here is not subject to these problems. We calculated the correlation between independent component time courses, with no prior global signal subtraction. The most significant results were as follow. The anticorrelation finding reported previously between the Default Mode and the dorsal attention network [5,11,42,43] was reproduced. The basal ganglia network was also found to be anticorrelated with the Default Mode, and correlated, to a similar degree, with the lateralised attention networks. Although we and others have shown that RSNs possess similar frequency characteristics, it must be the case that they are at most weakly coupled, or they would not be separable in functional connectivity analyses [47]. Correlation values observed here between component time courses are correspondingly low - in the range of 0.19 to 0.27 in magnitude - consistent with those observed in other studies [12]. Despite the fact that they are weak, their consistency across subjects is such that these results are highly significant. There have been suggestions that the thalamus, with involvement of the basal ganglia, may be responsible for instigating the task-independent deactivation of the Default Mode observed when subjects are posed cognitively demanding tasks [48]. This would be consistent with findings by Uddin et al., which have shown that correlations between homologous RSN structures in the cerebral hemispheres are preserved in a patient with complete commissurotomy, indicating that functional connectivity can be mediated by subcortical structures [49]. The hypothesis that the basal ganglia RSN represents the controller of fluctuations in the Default mode is supported by the results of the functional network connectivity analysis. In 18 out the 20 randomly composed groups of 13 subjects, the time course of the basal ganglia independent component preceded that of the Default Mode. Although this demonstrates that the hemodynamic response in the basal ganglia RSN consistently precedes that in the Default Mode, latency differences in the hemodynamic response functions [50] in both networks would have to be analysed and corrected for [51] before concluding that activation in the basal ganglia network precedes deactivation in the Default Mode. Possible differences between the precedence of neuronal activation and the measured MR response arising from hemodynamic shape and latency effects [52] preclude testing order hypotheses with this and other approaches such as Granger Causality Modeling.

Although RSNs show a maximum in frequency spectra in the range 0.01 - 0.04 Hz, this does not accurately reflect the frequency distribution of underlying neuronal fluctuations. The intrinsic autocorrelation of BOLD fMRI data has 1/f behaviour in the frequency domain [53], and low frequencies are cut off by sampling over a finite duration, leading to a low frequency peak. In fact, when the hemodynamic response function is deconvolved from RSN time courses prior to frequency analysis, the spectra of RSN components are essentially flat up to 0.1 Hz [27]. The analysis of RSN frequencies in this work serves two purposes, neither of which relate to the absolute frequencies observed in the spectra. The first is that differences between the compositions of RSN spectra can be observed. The frequency spectrum of the basal ganglia network is most similar in composition to that of the Default Mode, reinforcing the connection between the two networks. The second is that RSN spectra may be distinguished from components of physiological origin because their frequency distributions reflect the intrinsic convolution with the hemodynamic response function. This is the basis for the classifier used here, which can reliably distinguish RSNs from physiological components using frequency characteristics alone [54]. The basal ganglia RSN clusters clearly with other RSNs.

It is likely that the component which consists solely of the caudate (Figure 3) reflects activity relating to the inhibition of ocular saccades. As such, caudate activity is a task-specific response rather than an RSN. The caudate component is apparent in the fixation study, in which there was a point fixation condition, while no corresponding activity is apparent in the eyes closed study. The role of the caudate in saccadic eye movements is well documented [55-57]. Fluctuations in caudate activity in this capacity (which are a pre-requisite of their identification in an ICA) may arise due to phasic preparation of reflexive saccades and their voluntary inhibition. Ultimately controlled by the superior colliculus, saccades can be generated and inhibited by the input of the caudate nucleus, via efferent projections to the substantia nigra pars reticulata [57]. The oculomotor basal ganglia-thalamocortical circuit, includes, as origins of input to the caudate, frontal eye fields, dorsolateral prefrontal cortex and posterior parietal cortex (Brodmann's areas 8, 9&10 and 7, respectively) [36] all of which areas are apparent in this component. Frontal eye field neurons are known to fire during passive fixation [58] as was the condition in the fixation study. The oculomotor network yielded from the dorsal caudate seed in the Di Martino et al. analysis [31] is similar to the caudate component identified here in the fixation study. Our hypothesis that this is a task-related response to the suppression of ocular saccades is consistent with the use of a fixation condition in that work, and it not being observed in the many previous studies which have used the eyes closed condition [9,21-23,32].

Another area of application of this network is as a candidate marker for neuro- and psychopathologies involving the basal ganglia. In a parallel with attempts to use Default Mode activity as a diagnostic marker for Alzheimer's disease [8], deficits in the basal ganglia RSN may offer a marker for one or more of the diseases in which the basal ganglia are known to play a role. Parkinson's disease is a basal ganglia disorder characterized by the degeneration of dopaminergic neurons in the striatum. This has been shown to have opposing effects on the direct and indirect pathways in the striatum [59], with hyperkinesia as a result. Functional connectivity in the basal ganglia-cortical circuit has been demonstrated in this condition via synchronous oscillations between local field potentials in the basal ganglia and cortical EEG [60], the transmission to the basal ganglia of motor cortex electrostimulation in the monkey and rat [61] and oscillatory high-voltage spindles)[62]. Dopaminergic lesion in a rodent model for Parkinson's has been shown to lead to an increase in oscillatory synchronisation in the basal ganglia and increase in frequency and duration of high voltage spindle events)[62]. Similarly, excessive synchronisation of subthalamic nucleus neurons has been confirmed as a cause of movement slowing in Parkinsonism [63]. Dopamine-dependent changes in the functional connectivity between the basal ganglia and cortex have likewise been demonstrated [64]. The discovery of the fMRI manifestation of this functional connectivity will allow dysfunction in this system to be probed non-invasively, even if patients are not able to perform motor tasks appropriately.

## Conclusion

We report the existence of a resting state in the thalamus and basal ganglia, showing greatest activity in the putamen, pallidum, substantia nigra and subthalamic nucleus, with projections to transverse temporal gyrus and the supplementary motor areas. The network is consistent with the cortico-subcortical motor control circuit of the basal ganglia and is robustly reproducible across subjects, scanning parameters and common behavioural conditions for resting state studies. The frequency spectrum of this component peaks at 0.025 ± 0.007 Hz and is most similar in frequency composition to the Default Mode. Spectral features are similar to those of other resting state networks and distinct from physiological artefacts. Fluctuations in the basal ganglia network precede those in the Default Mode, with which it is anticorrelated. We posit that another independent component focussed in the caudate, which was observed in the fixation condition but not in the eyes closed condition, and which was recently reported as being associated with the resting state of the brain [31], is related to the suppression of ocular saccades.

The basal ganglia resting state network reported here offers the possibility to improve experiment design and analysis in fMRI studies of the striatum, and a possible window into disorders of the basal ganglia such as Parkinson's disease.

## Methods

### MR data acquisition, fixation study (3 T)

Twenty-six female right-handed subjects with no history of neurological or psychiatric disease and aged between 22 and 41 years (mean 26 ± 5 years) participated in the study, which was approved by the Ethics Committee of the Medical University of Vienna, with informed written consent. Magnetic resonance images were acquired with a 3 T Bruker Medspec S300 scanner (Bruker Biospin, Ettlingen, Germany) using a birdcage head coil. T_1_-weighted structural images were obtained with a 3D MPRAGE sequence with TE = 8 ms; flip angle = 15°; TA = 13 min. For the resting state run, which was of 5 minutes duration, subjects were asked to fixate on a point projected onto a screen mounted in the scanner bore, which they viewed via a mirror attached to the head resonator. They were instructed not to engage in organised thought and not to sleep. Compliance was established verbally on completion of the experiment. Resting state EPI data was acquired with 18 oblique slices (parallel to a line defined by the anterior and posterior commissures (ACPC)) of 4 mm thickness with a 1 mm nominal gap. Other imaging parameters were as follows: TE/TR = 28/1000 ms, a matrix size of 64 × 64 and a field of view of 21 × 25 cm^2 ^(left-right and anterior-posterior, respectively) yielding 3.3 × 3.9 × 4 mm^3 ^voxels, NR = 300, TA = 5 min. Imaging was prefaced by 10 s of dummy scans to ensure a steady state of longitudinal magnetisation.

### MR data acquisition, eyes closed study (4 T)

A second group of subjects was studied in the eyes closed condition. Fifteen subjects (8 males) with no history of neurological or psychiatric disease and aged between 19 and 56 years, mean 36 ± 12 years, participated in the study, which was approved by the Ethics Committee of the University of Trento, with informed written consent. Magnetic resonance images were acquired with a 4 T Bruker Medspec scanner (Bruker Medical, Ettlingen, Germany) using a birdcage-transmit, eight-channel receive head coil (USA Instruments, Inc., Ohio, USA). T_1_-weighted structural images were obtained using a 3D MPRAGE sequence with TE = 4 ms; flip angle = 7°; iPAT factor 2, TA = 5 min optimized for maximal contrast to noise ratio between grey and white matter at 4 T [65]. Subjects were asked to close their eyes during two resting state runs of 10 minutes duration each, and not engage in organised thought and not sleep (compliance established verbally as in the fixation study). EPI data were acquired along the ACPC line, with 37 oblique slices of 3 mm thickness with a 0.5 mm nominal gap, TE/TR = 33/2200 ms, a matrix size of 64 × 64 and 192 × 192 mm, yielding 3 × 3 × 3 mm voxels. 273 volumes plus 2 preparation scans were acquired in a 10 min session. Resting state runs were prefaced by a point-spread function acquisition to allow the correction of geometric distortions [66], which has been demonstrated to be effective at high field [67].

### Data Analysis

In the eyes closed study, distortion correction of EPI data was performed online using the point-spread function method as implemented in Siemens Distortion Correction WIP Version 2.5 [66]. Functional data acquired in both studies were preprocessed using SPM5 (motion correction, normalisation to the EPI template, no spatial or temporal smoothing). For both the fixation and control studies, Probabilistic Independent Component Analysis (PICA) was performed with MELODIC Version 3.05, part of FSL 4.0 [68]. The multi-session temporal concatenation group ICA approach was employed in order to find common spatial patterns amongst subjects without assuming similarity in temporal responses. Whilst the same approach is employed in the Group ICA of FMRI Toolbox (GIFT) [20], we used MELODIC to estimate component spatial maps in order both to facilitate comparison with the majority of studies which have looked for RSNs common to healthy subjects [21-23,32] and because probabilistic approach allows alternative hypothesis testing of the significance of activated voxels [18]. Downsampling of data is common in group ICA to reduce total data volume and to increase SNR. Here we analysed data at two resolutions - downsampled to 4 mm and also with no downsampling, the latter to try to resolve activation in small structures. In the PICA analysis masking was applied to exclude non-brain voxels. The data were de-meaned on a voxel-by-voxel basis, and the voxel-wise variance normalised. The number of components into which the data were decomposed was determined using the Laplace approximation to the Bayesian evidence for a probabilistic principal component analysis model implemented in MELODIC [18]. Estimated component maps were divided by the standard deviation of the residual noise. Statistical significance was attributed by fitting a mixture model to the histogram of intensity values [18]. To establish the reproducibility of responses the same analysis was applied to two equally sized, randomly selected, non-overlapping subgroups of 13 subjects in the fixation study; Subgroup 1, (mean age 25 ± 3 years) and Subgroup 2 (mean age 27 ± 6 years) (age difference not significant in student's t-test; P = 0.37).

Anatomical connectivity determines function [69]. The likelihood of brain regions being connected may be determined from diffusion weighted imaging data in an approach known as probabilistic diffusion tractography [41], which can provide useful insight into the roles of RSNs [21]. The probabilities of activation foci in the thalamus connecting to particular cortical regions were assessed using an open-access probabilistic diffusion tractography atlas http://www.fmrib.ox.ac.uk/connect/ based on the results of a study by Behrens et al. [41].

Data in the fixation study were additionally analysed with GIFT [20]. GIFT employs a temporal concatenation approach like that used in this MELODIC analysis, but also back-reconstructs single-subject spatial maps in addition to time courses. In our experience, the temporal responses of single subjects are better separated by GIFT, and we used these as the basis for analysis of temporal features, including characterisation of the frequency distributions of RSNs, the temporal relationships between RSNs (correlations between component time courses) and the attempt to distinguish RSNs and physiological components using temporal features. These further analyses were performed using MATLAB (Mathworks Inc, Natick, MA) routines developed in-house.

Frequency spectra were calculated from component time courses using Welch's averaged, modified periodogram spectral estimation method, using a Hamming window over periods of 64 s, with 50% overlap between segments. The peak frequency was calculated as the mean frequency over subjects at which the spectral power was a maximum.

To investigate correlations between component time courses (which may indicate functional relationships between networks [11,42]) the correlation coefficients between all possible pairs of component time courses were calculated. To avoid the possibility of introducing anticorrelations between networks [25,27,28], no global signal subtraction was performed. Fischer transformation was applied to single-subject correlation values (to normalise their distribution), and the null hypothesis of no correlation between the time courses tested with one-sample t tests at the P < 0.05 level.

An extension of the approach of correlating RSN time courses, termed Functional Network Connectivity [12], has been developed to allow the study of not only the correlation between networks, but also which leads and which lags in response. The functional network connectivity toolbox http://mialab.mrn.org/ was applied to GIFT results. In keeping with the identification of the peak in spectral power at 0.025 ± 0.007 Hz, correlations were assessed in the frequency range 0.01 to 0.4 Hz. Only lags between components reflecting the Default Mode and the basal ganglia resting state network were assessed, in view of the hypothesised connection between the two networks. A significance threshold of P < 0.05 was set for correlations. As an additional means to assess the reliability of calculated correlations and lags, the analysis was performed for each of 20 randomly-composed groups of 13 subjects from the population of 26 subjects.

Given differences in the frequency composition of RSN and physiological components (see [29] and Results section here), two features suggested themselves as the basis for a classifier. These have been labelled "Dynamic Range" - the difference in power between the maximum and the minimum of the distribution, and "Low to High Power Ratio" - the ratio of the integral of power in the region of the spectrum below 0.02 Hz to the total. Spectra were smoothed with MATLAB's digital filter function ([70] pp311-2), with the filter described by numerator coefficients [0.25 0.25 0.25 0.25] and with a denominator coefficient of 1, prior to the calculation of features, which were evaluated for each subject and component and plotted in the feature space. Discrimination thresholds were established using the iterative threshold selection algorithm described in Ridler and Calvard [71] in which the threshold starting point θ is the mean value of the entire bimodal distribution and the value of the threshold for the k^th ^iteration is

where m_s,k-1_, m_i,k-1 _are the means of the distributions inferior and superior to the threshold for the previous iteration. This was performed until θ_k _= θ_k-1_. To avoid introducing bias, the basal ganglia component was not included in the development of the classifier or the establishment of discrimination thresholds.

The most salient details of the two study groups and the analysis methods applied to each are shown in a data analysis flow chart, Additional File 4.

## Authors' contributions

SR conceived of the study, acquired the fixation fMRI data, performed the MELODIC ICA and FNC analyses, developed the interpretation and wrote the manuscript. US recruited and tested subjects and reviewed possible alternative interpretations of the network's function. GB acquired the eyes closed data, performed neuroanatomical labelling and assisted with interpretation. NS performed the GIFT analysis, frequency and correlation analyses and developed the classifier. JJ and LB guided the analysis of temporal and frequency features and the classifier and IKE, HB and EM contributed to interpretation of the network's function. All authors read and approved the final manuscript.

## Supplementary Material

Additional file 1 **Independent component for the basal ganglia RSN (MELODIC)**. This image is of the independent component for the basal ganglia identified in the MELODIC analysis, thresholded at P > 0.5 (downsampled data). Click here for file

Additional file 2 **Independent component for the basal ganglia RSN (GIFT)**. This image is of the independent component for the basal ganglia identified in the GIFT analysis (downsampled data). Click here for file

Additional file 3 **Hybrid simulations, ICA separation of spatially overlapping components**. This document contains text and images describing hybrid simulation showing that ICA is capable of separating circuits which overlap to some extent, but which also have non-overlapping elements and different temporal behaviour. Click here for file

Additional file 4 **Analysis flow chart**. This image shows the most important details relating to the two study groups and the analysis methods applied to each, presented as a flowchart. Click here for file
