# Supplementary figures and images for: A resting state network in the motor control circuit of the basal ganglia
Source: BMC Neurosci. 2009 Nov 23;10:137. doi: 10.1186/1471-2202-10-137 (PMC2785820; doi:10.1186/1471-2202-10-137)

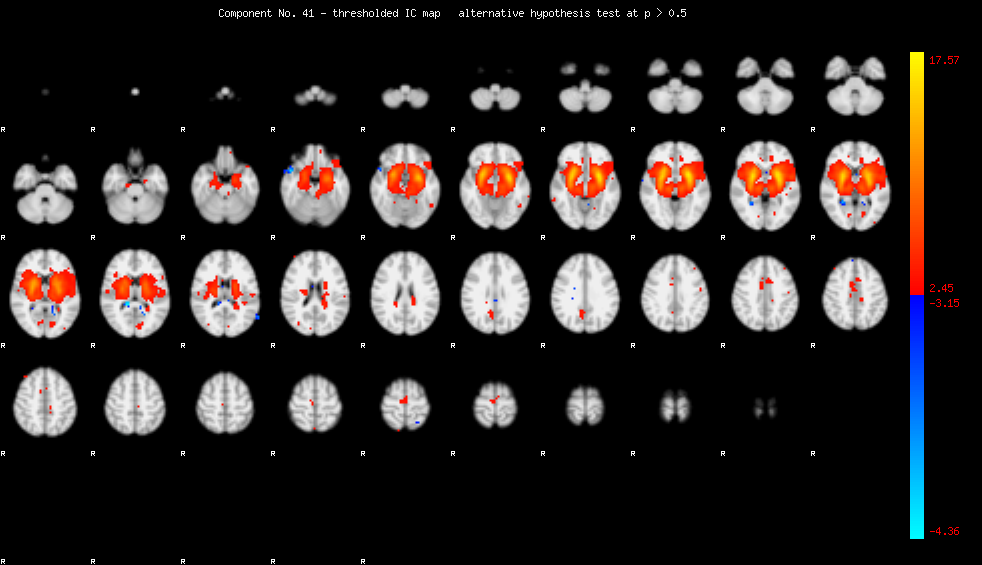

Supplement: Additional file 1 — Independent component for the basal ganglia RSN (MELODIC). This image is of the independent component for the basal ganglia identified in the MELODIC analysis, thresholded at P > 0.5 (downsampled data). [file 1471-2202-10-137-S1.PNG]

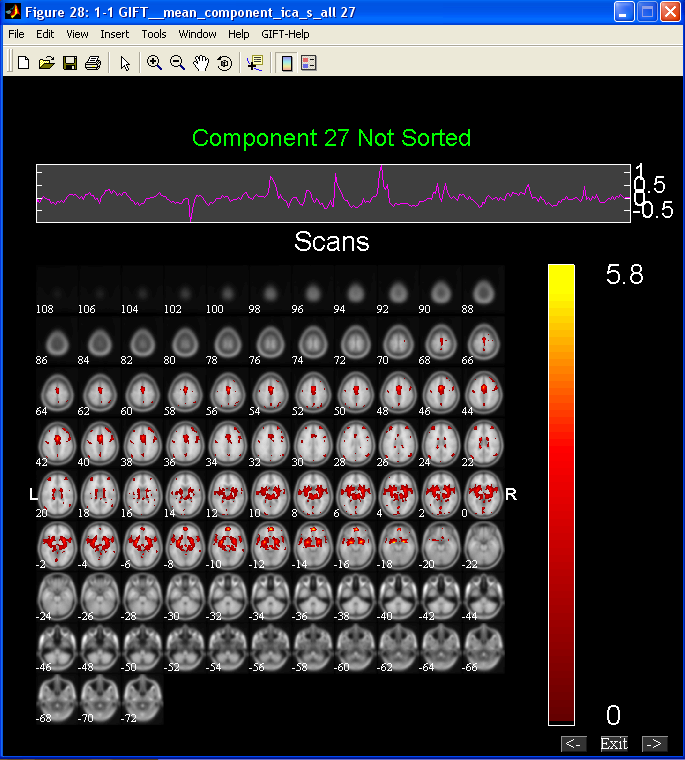

Supplement: Additional file 2 — Independent component for the basal ganglia RSN (GIFT). This image is of the independent component for the basal ganglia identified in the GIFT analysis (downsampled data). [file 1471-2202-10-137-S2.PNG]

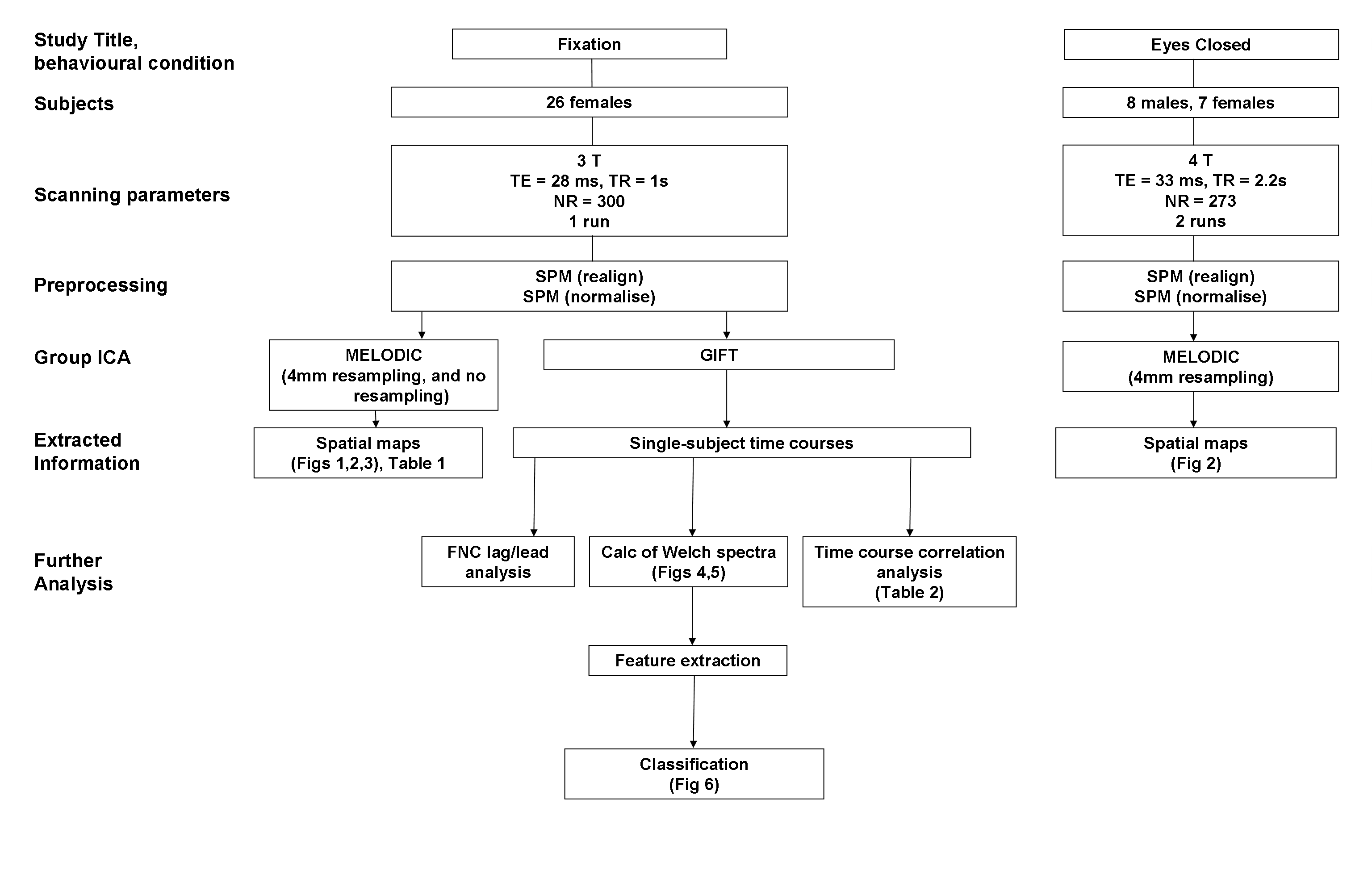

Supplement: Additional file 4 — Analysis flow chart. This image shows the most important details relating to the two study groups and the analysis methods applied to each, presented as a flowchart. [file 1471-2202-10-137-S4.PNG]
