# Supplementary material for: A resting state network in the motor control circuit of the basal ganglia
Source: BMC Neurosci. 2009 Nov 23;10:137. doi: 10.1186/1471-2202-10-137 (PMC2785820; doi:10.1186/1471-2202-10-137)
Supplement: Additional file 3 — Hybrid simulations, ICA separation of spatially overlapping components. This document contains text and images describing hybrid simulation showing that ICA is capable of separating circuits which overlap to some extent, but which also have non-overlapping elements and different temporal behaviour. [file 1471-2202-10-137-S3.PDF]

The possibility of separating partially overlapping RSNs, e.g. corresponding to functionally distinct basal ganglia circuits.

Sinusoidal signal sources were introduced into hybrid data (a time-series created from replication of a single EPI volume) in brain regions indicated in Figure 1 with characteristics listed in the table below and the addition of 5% Gaussian noise. Regions 1 and 2 in network (a) did not overlap with any regions in network (b), but regions 3 and 4 were identical in the two networks. The frequencies of the fluctuations introduced were distinct (0.1 and 0.15 Hz for networks (a) and (b) respectively). If ICA is capable of separating the signal sources (a) and (b), in which some sources locations are identical, then it could be expected to separate basal ganglia networks in which some, though not all, elements overlap either because they are involved in both networks or because – although spatially distinct - they cannot be resolved.

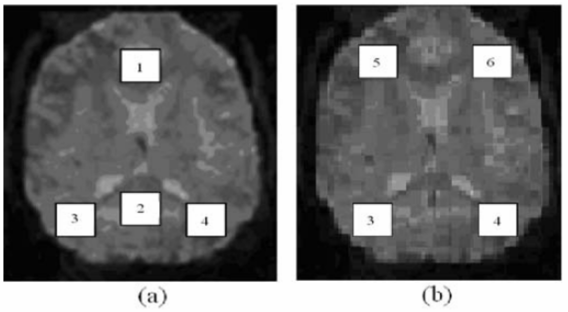

RSN Label	Amplitude (a.u.)	Frequency (Hz)
(a)	0.03	0.1
(b)	0.02	0.15

Figure 1: Hybrid data used for simulation to test the extent to which spatially overlapping networks can be distinguished by ICA. The regions used were centered at the locations indicated by the numbers above, but were smaller than those shown in the figure.

The components illustrated in Figure 2 show that ICA can indeed separate these sources and accurately identify both the source locations and frequencies of the fluctuations without mixing. We would therefore expect our analysis to be able to identify distinct thalamo-cortical networks despite regions of source overlap, if these were present. The non-observation of other parallel circuits in this study is more likely related to those networks not showing spontaneous fluctuations, or any fluctuations being below the study sensitivity.

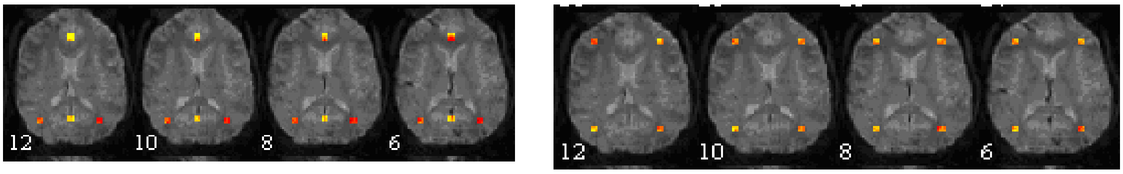

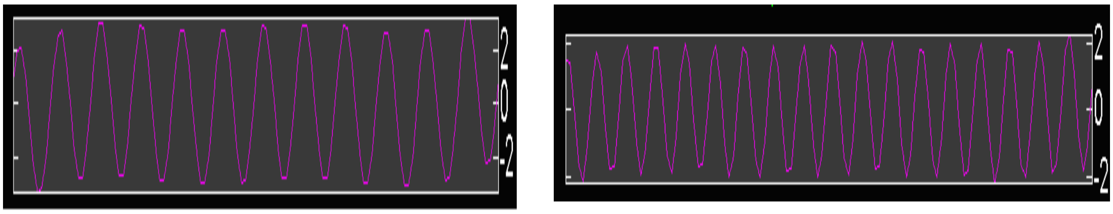

Figure 2: ICA results (using GIFT). Networks (a) and (b) are identified in two components (left and right respectively) with no mixing of sources, and with accurate reproduction of the frequency signatures of the fluctuations introduced.

A functional connectivity analysis (FCA) would also be expected to separate these sources if ROIs 1, 2, 5 or 6 were used as seed regions, but the sensitivity would be reduced and networks would become highly mixed if the selected seeds regions were in ROIs 3 or 4. The results of these tests are consistent with literature findings, e.g. that there is substantial mixing of respiration rate-related physiological fluctuations and Default Mode Network fluctuations in FCA (Birn et al., 2006) but not ICA (Birn et al., 2008), and that some brain regions (such as the thalamus) appear in multiple networks.
